# Frequency of Pregnancy-Associated Cancer: A Systematic Review of Population-Based Studies

**DOI:** 10.3390/cancers12061356

**Published:** 2020-05-26

**Authors:** Michela Dalmartello, Eva Negri, Carlo La Vecchia, Giovanna Scarfone, Barbara Buonomo, Fedro A. Peccatori, Fabio Parazzini

**Affiliations:** 1 Department of Clinical Sciences and Community Health, University of Milan, 20122 Milan, Italy; michela.dalmartello@unimi.it (M.D.); carlo.lavecchia@unimi.it (C.L.V.); fabio.parazzini@unimi.it (F.P.); 2 Department of Clinical and Biological Sciences, University of Milan, 20122 Milan, Italy; eva.negri@unimi.it; 3 Department of Obstetrics, Gynecology and Neonatology, University of Milan, Fondazione IRCCS Ca’ Granda Ospedale Maggiore Policlinico, 20122 Milan, Italy; giovanna.scarfone@policlinico.mi.it; 4 Fertility and Procreation Unit, Gynecologic Oncology Program, European Institute of Oncology IRCCS, 20141 Milan, Italy; barbara.buonomo@ieo.it

**Keywords:** cancer, pregnancy, population-study, frequency

## Abstract

Despite numerous available resources of evidence, the results about the frequency of pregnancy-associated cancer (PAC) still show poor comparability due to dissimilarities in the study design and methodology, inclusion criteria, incoherent duration of follow-up and a heterogeneous reference population. We conducted a systematic review of population-based studies on PAC published up to December 2019, to provide updated research on this topic, highlighting strengths and limitations. Of the 24 papers included, 11 considered all types of tumors and 13 dealt with specific types of cancer. Differences in the procedures for estimating the frequency of PAC emerged even among population studies. However, we found consistent results for overall frequency of PAC— around 1/1000 pregnancies. Our review suggests that about 25% of PAC cases are diagnosed during pregnancy, confirming the hypothesis of an excess of diagnosis in the postpregnancy period. Sparse and inconsistent results were found regarding a potential increase in the frequency of PAC over calendar years. Alignments in the strategy to identify PAC are needed to overcome methodological weaknesses.

## 1. Introduction

Malignancies occurring in association with pregnancy pose major challenges for patients, their physicians and health care systems. Pregnant patients diagnosed with cancer need a multidisciplinary approach with the involvement of different specialists. Both maternal and fetal health should be considered, and specialized management strategies should be implemented to ameliorate clinical results. Unmet needs in health care system organizations may become evident when incidence and prevalence of a specific condition is over- or under-evaluated. Thus, it is of utmost importance to quantify the burden of cancer in pregnancy.

Pregnancy-associated cancer (PAC) complicates approximately 1/1000 pregnancies [1]. However, there is a considerable variation in incident and prevalent cancer types in different populations. A comparison of the available data is complex because of differences in inclusion criteria and in data extraction methods, incoherence in the definition of follow-up, and dissimilar reference populations. PAC is often reported to be increasing, and mainly attributed to the rising childbearing age of the mothers, particularly in high-income countries [1,2]. Nonetheless, this has not been adequately addressed, and the relationship between the suspected increase and ageing of mothers is still controversial.

Most evidence on this topic originated from single hospital experiences, i.e., from data gathered in referral centers. Hence, questions regarding the generalizability of these results to the population remain. Population-based studies may provide more reliable estimates, given a smaller impact of selection and recall bias [2].

The aim of this work is to review and provide updated evidence on this topic, highlighting differences, strengths and limitations among population-based studies on PAC.

## 2. Methods

### 2.1. Search Strategy and Study Selection

A PICO (population, intervention, comparison and outcome) design structure was used to develop the study question and the inclusion/exclusion criteria. Published articles providing results on PAC were identified from the Medline database through PubMed, using the string “(((pregnancy [Title/Abstract] OR pregnant [Title/Abstract])) AND (cancer [Title/Abstract] OR neoplasm* [Title/Abstract]) AND (incidence [Title/Abstract])) OR (("Pregnancy Complications, Neoplastic/epidemiology" [Mesh] OR "Pregnancy Complications, Neoplastic/statistics and numerical data" [Mesh])).” A similar strategy was applied to the search by Embase, using the string “(pregnancy: ti,ab OR pregnant: ti,ab) AND (cancer: ti,ab OR neoplasm: ti,ab OR neoplasms: ti,ab) AND (incidence: ti,ab OR ’cancer incidence’/exp) AND [English]/lim AND [embase]/lim AND ([embase]/lim OR [medline]/lim) AND ([embase]/lim OR [medline]/lim OR [pubmed-not-medline]/lim)”. Research was restricted to papers published in English and published up to 18 December 2019. Additional pertinent reports were extracted by checking the reference lists of the retrieved articles and selected reviews. The work was completed and reported according to the PRISMA guidelines for the reporting of systematic reviews. Two authors independently selected the articles and retrieved the potentially relevant ones. Discrepancies were resolved by discussion.

We defined PAC as tumors diagnosed during pregnancy or postpregnancy (no restriction of the postpregnancy period was used). Tumors derived from placental tissues (e.g., choriocarcinoma) are by definition gestational tumors; thus, they are not considered PAC by most authors and were not included in this review.

Studies were excluded if they were (i) single hospital studies or survey studies; (ii) letters to editors, commentaries, conference abstracts. Studies performed on the same population but considering different periods of observation and different sources were all included [2,3,4,5]. Five studies performed on the same population, but focusing on different types of PAC, [4,5,6,7,8,9,10] were included.

Moreover, studies were included if they estimated incidence or reported both the numbers of PACs (overall or for specific type of cancer) and the total number of pregnancies/deliveries/live births considered as population. Pregnancy included every case diagnosed during the 9 months of gestation, irrespective of its duration, while delivery included both stillbirths and live births. Live birth was defined as “the complete expulsion or extraction from its mother of a product of conception, irrespective of the duration of the pregnancy, which, after such separation, breathes or shows any other evidence of life” [11].

### 2.2. Data Extraction

For each study, the following data were extracted: 1. general characteristics of the study (first author, year of publication and country); 2. study design and characteristics (type of the study, data source, period of the study, procedure for PAC identification); 3. quantification of PAC (definition of PAC, size and type of population, number of PACs, type, rate and distribution during pregnancy) and 4. other data (assessment of trend during the observation period, and ranking of the most frequent PAC for studies focusing on PAC overall). When some statistics were not present, they were derived from available data, where possible.

## 3. Results

**Figure 1:**
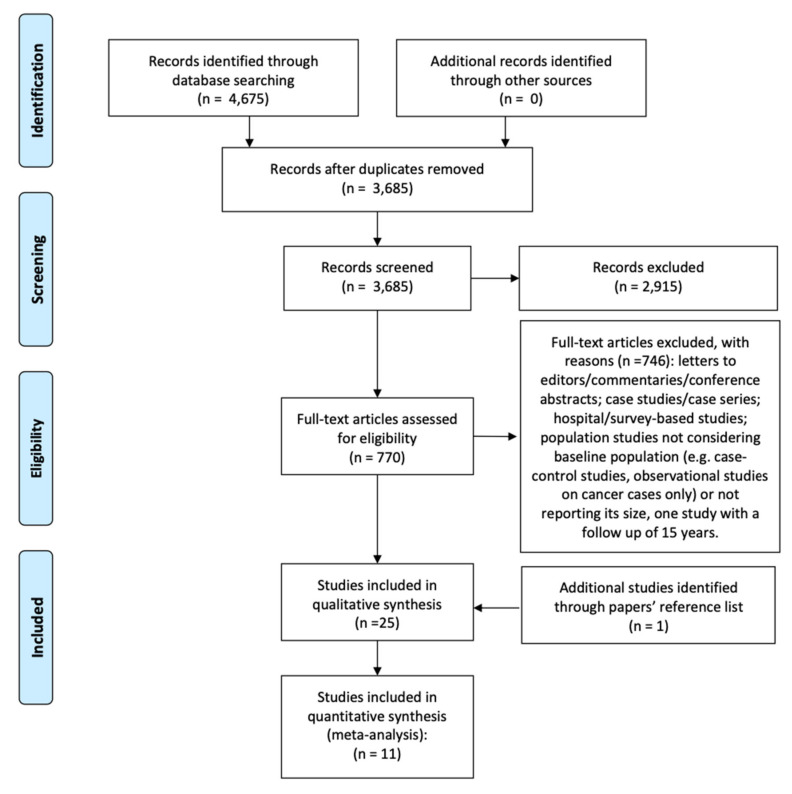
PRISMA flowchart summarizing the process for the identification of eligible articles.

We identified 4675 articles, and 3685 of them remained after the exclusion of duplicates. Among them, we identified 770 articles dealing with PAC. After application of inclusion–exclusion criteria, 23 articles were selected. From the reference list of the selected papers, we found another article [4]. Of the 24 papers included in the review, 11 considered all types of tumors and 13 dealt with one specific type of cancer (Figure 1).

### 3.1. General Characteristics

General characteristics and study design information from the 24 papers are presented in Table 1. The selected papers were published from 1995 to 2019, most of them after 2010. Of these, 13 were conducted in the USA [4,5,6,7,8,9,10,12,13,14,15,16,17], 7 in Europe [18,19,20,21,22,23,24], mainly in the northern Europe, and 4 in Australia [2,3,25,26]. The majority of the studies were based on national registries. Five studies relied on a database covering 20% of country community hospital inpatient stays [12,13,15,16,17], and one used data from two multicentric studies [14].

Among the registry-based studies, ten used data from a linkage between a hospital discharge registry, a maternal/birth registry and a cancer registry [2,3,4,6,7,8,9,10,25,26]. Two studies linked a hospital discharge registry and a cancer registry [18,19] and four studies linked cancer registry with a maternal/birth registry [14,20,21,22]. Seven studies relied only on a hospital discharge database [12,13,15,16,17,23,24], and one study collected the information regarding baseline population size from national statistics [26].

A total of 20 studies identified pregnancies/deliveries/live births, and then identified cancer cases using the same data source or by record linkage with external database [2,3,4,5,6,7,8,9,10,12,13,14,15,16,17,18,22,23,24,25]. Three studies started identifying cancer cases and then pregnant women within the same population [19,20,26]. One study did not report the procedure of PAC identification [21]. Among the 20 studies which firstly selected the baseline population, five (out of the seven using only a hospital discharge registry), restricted information to the time of delivery, thus obtaining cancer cases present at time of delivery [12,13,15,16,17].

### 3.2. Definition and Estimated Frequency of PAC

Table 2 reports details about PAC frequency calculation. An amount of 5 studies estimated PAC among pregnancies [14,20,23,24,26], 16 among deliveries [2,3,4,5,6,7,8,9,10,12,13,15,16,17,19,22] and 3 among live births [18,21,25]. Most studies estimated PAC frequency from their baseline population. In contrast, two studies identified PAC among pregnancies and estimated the rate using the number of births as a denominator [20,25]. Population sizes ranged from 679,736 to 11,846,300 subjects for studies on deliveries, from about 33,000 to 6,111,111 for studies on pregnancies and from 1,309,501 to 4,580,005 for studies on live births.

PAC was defined in the majority of the studies [2,3,4,5,14,18,19,20,21,22,23,24] as cancer occurring during pregnancy and up 1 year [2,3,4,5,6,7,8,9,10,14,20,21,22,23,24,25] or 2 years [18,19] after delivery. Two studies focused on pregnancies used different follow-up for pregnancy ending with abortions vs. delivery: 3 months vs. 9 months prior to pregnancy end and up to 12 months after [23,24]. Five studies considered only the pregnancy period [12,13,15,16,17].

The above-mentioned studies defined the follow-up period around the delivery date. However, one paper defined the follow-up period around the time of cancer diagnosis or treatment [26].

Most papers considered invasive types of PAC. Only two studies considered also non-nvasive tumors [25,26] and three studies did not report this information [20,21,22].

The number of PAC ranged from 189 to 7890. The number of cases ranged from 573 to 1161 for breast cancer, from 434 to 2944 for cervical cancer, and from 412 to 577 for melanoma. A study on colorectal cancer identified 134 cases; the study on thyroid cancer identified 595 cases; the studies on non-Hodgkin’s lymphoma identified 427 cases and another one on Hodgkin’s lymphoma identified 638 cases.

**Figure 2:**
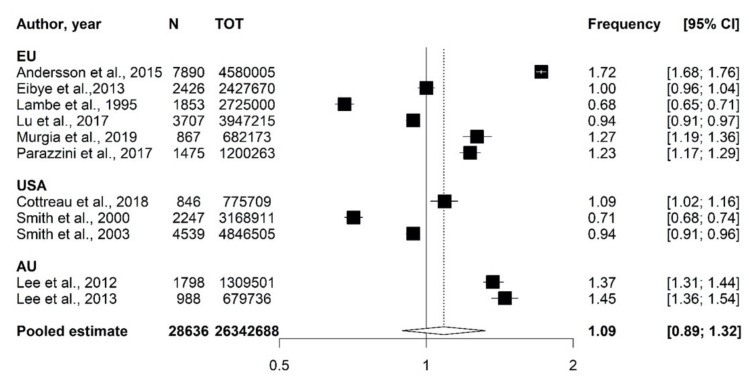
Frequencies of pregnancy-associated cancer (PAC): estimates by study and overall estimate and related 95% confidence intervals.

Frequencies of PAC ranged between 0.90 and 1.27/1000 pregnancies, between 0.71 and 1.72/1000 deliveries, and 0.68 and 1.72/1000 live births. Estimates by study and country are reported in Figure 2. The overall estimate was 1.09 (95% confidence interval = 0.89–1.32). 

For specific types of cancer, estimates were more variable (Table 2).

With reference to the distribution of PAC diagnosis prior or after birth, five studies did not consider the postpregnancy period in the identification of PAC [12,13,15,16,17]. One study did not report the distribution during pregnancy and postpregnancy [3]. Most of the remaining studies found a higher prevalence of PACs during the postpartum period (rates between 53.3% and 99.3%, mean = 77.0%). The same tendency was noted among studies concerning specific PACs, with rates ranging between 63.8% and 91.5% in the postpartum period.

Most of the studies on PACs reported the most frequent types of tumor [2,3,4,5,14,18,20,21,22,23,24] (Table S1). In a Swedish study, the most common tumors were breast, melanoma and cervical cancer. In a Danish study, melanoma ranked first, followed by cervical and breast cancer. Italian studies reported breast and thyroid as the most common cancer types, followed by skin cancer [23] or lymphoma [24]. In the studies for the USA, the two most frequent tumors were breast and thyroid cancer, followed by cervical cancer [4,5] or melanoma [14]. In the Australian ones, the most common cancer was melanoma, followed by breast and thyroid/endocrine tumors.

The length of the observation period (i.e., calendar years) varied across studies, from 6 years [4] to 40 years [19] (median = 10 years) (Table S2).

Six studies investigated the evolution of the incidence of PAC overall over calendar years. Three studies did not find a clear trend [4,23,24], while three studies found an increase in the incidence [2,14,20]. Two of them also investigated the relationship of the trend with a possible increase in age of mothers [2,20] and found that the trend was present even after adjusting for maternal age.

Sparse results were found also for specific types of tumors. Three studies assessed the trend for breast cancer [12,17,19]: two of them found an increase in incidence [17,19], one of them also after adjusting for age of the mother [19].

No trend in incidence was found for cervical cancer [7,13]. An increase was found for Hodgkin lymphoma [16] while no trend was found for non-Hodgkin lymphoma [15]. The two studies on melanoma gave inconsistent results [8,25].

## 4. Discussion

Most studies on PAC are based on case reports, case series or hospital-based data. A few studies on registries gave a population-based overview of the phenomenon, with generally high quality and completeness and hence lower selection bias. The aim of this work was to add more evidence on PAC by reviewing all population-based studies on this topic. Our results show that estimates of the incidence of PAC still maintain a level of sparseness even among population studies. Moreover, we could present and compare estimates across different geographical areas.

Cancer registries are a valid source for identifying incident cancer in the population, but their linkage to pregnancy/birth registries presents difficulties and uncertainties. In contrast, hospital-discharge records lack the identification of incident cancer cases (that have to be defined by a proper strategy), but can provide detailed information on the quality of care, on the adherence to diagnostic therapeutic pathways, covering all the follow-up period needed for identification of PAC. Some studies reported a satisfactory validation of hospital-discharge records [24] while others [3,5] highlighted over- or under-reporting on the basis of the algorithm of cancer identification. Some of birth and neonatal outcomes of PAC can only be addressed via birth/fertility registries. These registries are restricted to women whose pregnancy ended up in a delivery.

Estimates of the incidence of PAC benefits from the linkage of several databases [5]. For example, using both hospital discharge forms and cancer registries enhanced the identification of cases as the diagnosis of cancer may occur in outpatient settings. Using both hospital discharge registries and birth/fertility registries may shed light on all pregnancy outcomes in PAC cases, from abortions to live births outcomes.

Most of studies estimated PAC among deliveries, but in some of those was effectively possible to consider pregnancies. Restricting the reference population (pregnancy vs. deliveries vs. live births) underestimates the number of cases diagnosed during pregnancy. In addition, many registries do not include early miscarriages: not considering women whose pregnancy ended up with an abortion is an unsurmountable weakness of registry-based studies. This reduces the observed occurrence rate for cancer diagnosed in the early prenatal period, leading also to a lack of quantification of the pregnancy terminations due to PAC [18].

Despite generally pointed out as a frequent comparability problem among the studies about PAC, we found satisfactory coherent findings in most of the studies in focusing on invasive cancer cases with rates being around 1/1000 with a relative strict uncertainly range of 0.9–1.3.

The definition of PAC is homogenous in most studies, as a cancer diagnosed during pregnancy up to 1 year after birth. The inclusion of the postpartum period in the definition of PAC mainly derives from the fact that the origins of these cases may be ascribable prior to delivery. Most PAC were diagnosed in the postpartum. The higher number of PAC in postpregnancy implies the importance of considering a standardized period for this part of the follow-up to provide comparable estimates.

Our review suggests that about 25% of PAC cases are diagnosed in pregnancy. Hormones and growth factors in pregnancy may accelerate tumor growth [27]. However, the role of pregnancy in hormonal related carcinogenesis is out of the scope of this review and it should be investigated focusing more on analytical than descriptive epidemiological studies. Otherwise, the increased contacts with health care service during pregnancy may lead to a diagnosis anticipation. The antenatal and postnatal care visits and examinations may increase cancer detection. On the other hand, clinicians may be more reluctant to perform potentially harmful diagnostic procedures during pregnancy. This would result in a delayed diagnosis. Apart from diagnostic reluctance in pregnancy, one explanation of the postpartum rebound effect [2,5] was that the pregnancy itself brings physiological changes that may mask the existence of a tumor, leading to a delay in diagnosis. This may be particularly true for breast and thyroid cancer, due to changes in the mammary and thyroid glands, and melanoma for hyperpigmentation and naevi growth in pregnant women [18].

Moreover, while less aggressive cancers are more likely to be diagnosed lately, more aggressive PAC that are early diagnosed in pregnancy may lead to pregnancy termination [5]. Some authors, in fact, reported lower incidence than expected, even when considering the underreporting of PACs due to the lack of information on early pregnancy loss [18]. We should also consider the ‘healthy mother effect’: women affected by subclinical cancers are less likely to become pregnant.

Only a few studies considered the incidence of specific PAC, because generally the studies focusing on a specific cancer aimed to address outcomes, treatment and characteristics of cases. Thus, these studies were mainly case-controls or observational studies on PAC population only. Most studies agreed that the ranking of specific types of tumors was similar between pregnant and not pregnant women of the same age. In most studies, breast, thyroid cancer and melanoma were the most common cancer sites. The studies reporting cervical cancer among the most common [18,20,21,22], included data since the 1960–70’s, when cervical screening was not routinely performed in the general population. Moreover, the differences in the incidence of specific cancer types observed across countries may be due to different cancer incidences in the respective general populations: indeed, countries with a higher incidence of melanoma in the general population (including nonpregnant young women) have a higher incidence of PAC [25].

In one study, cancer incidence was higher than that expected among women of similar age [2].

The frequency of PAC cases has been suggested to be increasing, mirroring the increase in maternal age observed in all the developed countries during the last decades. Only a few studies analyzed trends in the frequency of PAC over years. The studies that found an increase in the incidence of PAC were generally the longest, and no trend was found among studies with a follow-up shorter than 10 years. Aspects like country-specific trends in the childbearing age and fertility rates are considered when interpreting these data. Mean maternal age has increased during the last 20 years of about 2 years in most developed countries [1,2,28]. This increase may be reflected only in a limited way in the frequency of PAC. Moreover, when controlling for the age of the mothers, the trend was still present. In addition, improved diagnostic techniques and health care services among recent years may partially or largely account for the observed trend.

## 5. Conclusions

In conclusion, available data about the frequency of PAC still have a degree of uncertainty and inconsistence. Some common traits and findings can nevertheless be identified, with an overall frequency of around 1/1000 pregnancies. This review does not show an increasing frequency of PACs over time, but it confirms previously reported incidence estimates. According to all the reviewed studies, we reported that PACs are mostly detected in the postpregnancy period.

In order to provide an optimal health care for women with PAC, there is a need for accurate, country-specific and cancer site-specific estimates, regarding the expected occurrence of PAC cases and the related maternal, neonatal and children outcomes. It is important to develop national programs to create registries where data about cancer diagnosis and management and information about pregnancy outcomes (abortion/deliveries/live births) are easily accessible. Further effort is needed to homogenize strategies and methods addressing the identification of PAC, and specific methodological issues (i.e., the coverage of the appropriate target population) and research topics (i.e., the assessment of the trends over time) require additional investigations.

## Figures and Tables

**Table 1:** Characteristics of the study, sources and procedure of identification of pregnancy-associated cancer (PAC). Studies on PAC overall and on specific PAC.

Author	Country	Years	Source	Type					PAC Selection:
Studies on PAC Overall									
				H	C	B	A	O	Procedure
Andersson et al., 2015 [18]	EU: Sweden	1963–2007	Swedish Multigeneration Registry, Swedish Cancer Registry	X	X				1.Births from H 2.PAC, linking 1 to C
Cottreau et al., 2019 [14]	USA	2001–2013	Cancer Research Network (CRN), Medication Exposure in Pregnancy Risk Evaluation Program (MEPREP) ^a^		X	X			1.Deliveries from B 2.PAC, linking 1 to C
Eibye et al.,2013 [20]	EU: Denmark	1977–2006	Danish Cancer Registry, Medical Birth Registry, Registry for Induced Abortions, National Patient Registry		X	X	X		1.Cancer cases from C 2.PAC, linking 1 to B and A
Lambe et al., 1995 [21]	EU: Sweden	1960–1990	Swedish Fertility Registry, Swedish Cancer Registry		X	X			NR
Lee et al., 2012 [2]	AU: New South Wales	1994–2008	Perinatal Data Collection (PDC), Central Cancer Registry (CCR), Admitted Patient Data Collection (APDC)	X	X	X			1.Deliveries from B 2.PAC, linking 1 to C and H
Lee et al., 2013 [3]	AU: New South Wales	2001–2008	Perinatal Data Collection (PDC), Central Cancer Registry (CCR), Admitted Patient Data Collection (APDC)	X	X	X			1.Deliveries from B 2.PAC, linking 1 to C (H used only for estimating outcomes)
Lu et al., 2017 [22]	EU: Sweden	1973–2012	Swedish Medical Birth Registry (MBR), Swedish Cancer Registry		X	X			1.Deliveries from B 2.PAC, linking 1 to C
Murgia et al., 2019 [23]	EU: Italy	2003–2015	National discharge Registry (SDO)	X					1.Deliveries/abortions from H 2.PAC among 1
Parazzini et al., 2017 [24]	EU: Italy	2001–2012	National discharge Registry (SDO)	X					1.Deliveries/abortions from H 2.PAC among 1
Smith et al., 2001 [4]	USA: California	1992–1997	Vital statistics birth/patient discharge (VS/PDD), linking: national discharge forms, birth certificates, death certificates	X		X			1.Deliveries from H-B already linked 1.PAC among 1
Smith et al., 2003 [5]	USA: California	1991–1999	Vital statistics birth/patient discharge (VS/PDD), linking: national discharge forms, birth certificates, death certificates; California Cancer Registry (CCR)	X	X	X			1.Deliveries from H-B already linked 2.PAC, linking 1 to C
**Studies on Specific PAC**									
				H	C	B	A	O	Procedure
Abenhaim et al., 2012 [12]	USA	1999–2008	United States Healthcare Cost and Utilization Project, Nationwide Inpatient Sample (HCUP-NIS) ^b^	X					1.Deliveries from H 2.PAC among 1 at delivery
Al-Halal et al., 2012 [13]	USA	1999–2008	United States Healthcare Cost and Utilization Project, Nationwide Inpatient Sample (HCUP-NIS) ^b^	X					1.Deliveries from H 2.PAC among 1 at delivery
Andersson et al., 2009 [19]	EU: Sweden	1963–2002	Swedish Multigeneration Registry, Swedish Cancer Registry	X	X				1.Cancer cases from C 2.PAC, linking 1 to H
Bannister-Tyrrell et al., 2014 [25]	AU: New South Wales	1994–2008	Perinatal Data Collection (PDC), Central Cancer Registry (CCR), Admitted Patient Data Collection (APDC)	X	X	X			1.Deliveries from B 2.PAC, linking 1 to C and H
Dahling et al., 2009 [6]	USA: California	1991–1999	Vital statistics birth/patient discharge (VS/PDD), linking: national discharge forms, birth certificates, death certificates; California Cancer Registry (CCR)	X	X	X			1.Deliveries from H-B already linked 2.PAC, linking 1 to C
Dalrymple et al., 2005 [7]	USA: California	1991–1999	Vital statistics birth/patient discharge (VS/PDD), linking: national discharge forms, birth certificates, death certificates; California Cancer Registry (CCR)	X	X	X			1.Deliveries from H-B already linked 2.PAC, linking 1 to C
El-Messidi et al., 2014 [15]	USA	2003–2011	United States Healthcare Cost and Utilization Project, Nationwide Inpatient Sample (HCUP-NIS) ^b^	X					1.Deliveries from H 2.PAC among 1 at delivery
El-Messidi et al., 2015 [16]	USA	2003–2011	United States Healthcare Cost and Utilization Project, Nationwide Inpatient Sample (HCUP-NIS) ^b^	X					1.Deliveries from H 2.PAC among 1 at delivery
Ives et al., 2005 [26]	AU: Western Australia	1982–2000	Western Australia Data Linkage System (WALDS)	X	X	X			1.Cancer cases from H-B-C already linked 2.PAC among 1
O’Meara et al., 2005 [8]	USA: California	1991–1999	Vital statistics birth/patient discharge (VS/PDD), linking: national discharge forms, birth certificates, death certificates; California Cancer Registry (CCR)	X	X	X			1.Deliveries from H-B already linked 2.PAC, linking 1 to C
Rodriguez et al., 2008 [9]	USA: California	1991–1999	Vital statistics birth/patient discharge (VS/PDD), linking: national discharge forms, birth certificates, death certificates; California Cancer Registry (CCR)	X	X	X			1.Deliveries from H-B already linked 2.PAC, linking 1 to C
Shechter Maor et al., 2018 [17]	USA	1999–2012	United States Healthcare Cost and Utilization Project, Nationwide Inpatient Sample (HCUP-NIS) ^b^	X					1.Deliveries from H 2.PAC among 1 at delivery
Yasmeen et al.,2005 [10]	USA: California	1991–1999	Vital statistics birth/patient discharge (VS/PDD), linking: national discharge forms, birth certificates, death certificates; California Cancer Registry (CCR)	X	X	X			1.Deliveries from H-B already linked 2.PAC, linking 1 to C

H: Hospital Discharge Registry; C: Cancer Registry; B: Birth/Fertility Registry; A: Abortion Registry; O: Other. ^a^ Data from five health plans participating in both the Cancer Research Network (CRN) and the Medication Exposure in Pregnancy Risk Evaluation Program (MEPREP). ^b^ Database sampling 20% of American community hospital inpatient stays.

**Table 2:** Definition and estimated frequency of pregnancy-associated cancer (PAC). Studies on PAC overall and on specific PAC.

Author	Type of Population	Size of Population	Type of PAC	Size (Rate on 1000) of PAC	Length of Risk Period Before–After Pregnancy End	Size (%) PAC in Pregnancy	Size (%) PAC in Postpregnancy
Studies on PAC Overall							
Andersson et al., 2015 [18]	LB	4,580,005	Invasive	7890 (1.72)	9 m–24 m	2042 (25.9%)	5848 (74.1%)
Cottreau et al., 2019 [14]	P	775,709	Invasive ^a^	846 (1.09)	9 m–12 m	243 (28.7%)	603 (63.8%)
Eibye et al.,2013 [20]	P	2,427,670	n.r. ^a^	2426 (0.896)	9 m–12 m	572 (23.6%)	1854 (78.3%)
Lambe et al., 1995 [21]	LB	2,700,000 ^b^	n.r. ^a^	1853 (0.68)	9 m–12 m	428 (23.1%)	1425 (76.9%)
Lee et al., 2012 [2]	D	1,309,501	Invasive ^a^	1798 (1.373)	9 m–12 m	495 (27.5%)	1785 (99.3%)
Lee et al., 2013 [3]	D	679,736	Invasive ^a^	988 (1.454)	9 m–12 m	n.r.	n.r.
Lu et al., 2017 [22]	D	3,947,215	n.r. ^a^	3707 (0.939)	9 m–12 m	984 (26.5%)	2723 (73.5%)
Murgia et al., 2019 [23]	P	682,173	Invasive	867 (1.27)	9 m (p.)/3 m (a.)–12 m	131 (15.1%)	736 (84.9%)
Parazzini et al., 2017 [24]	P	1,200,263	Invasive	1475 (1.23)	9 m (p.)/3 m (a.)–12 m	300 (20.3%)	1175 (79.7%)
Smith et al., 2001 [4]	D	3,168,911	Invasive	2247 (0.71)	9 m–12 m	1049 (46.7%)	1198 (53.3%)
Smith et al., 2003 [5]	D	4,846,505	Invasive	4539 (0.94)	9 m–12 m	1315 (36.4%)	2888 (63.6%)
**Studies on Specific PAC**							
Abenhaim et al., 2012 [12]	D	8,826,137	Invasive Breast	573 (0.065)	9 m–n.c.	573 (100%)	n.c.
Andersson et al., 2009 [19]	D	4,156,190	Invasive Breast	1161 (0.279)	9 m–24 m	99 (8.5%)	1062 (91.5%)
Bannister-Tyrrell et al., 2014 [25]	LB ^c^	1,309,501	Melanoma ^d^	577 (0.451)	9 m–12 m	195 (33.8%)	382 (66.2%)
Al-Halal et al., 2012 [13]	D	8,826,137	Invasive Cervix	294 (0.03)	9 m–n.c.	294 (100%)	n.c.
Dahling et al., 2009 [6]	D	4,848,505	Invasive Colorectal	134 (0.028)	9 m–12 m	36 (26.9%)	103 (76.9%)
Dalrymple et al., 2005 [7]	D	4,846,505	Invasive Cervix	434 ^e^ (0.12)	9 m–12 m	132 (31.3%)	298 (68.7%)
El-Messidi et al., 2014 [15]	D	7,917,453	Invasive non-Hodgkin’s	427 (0.054)	9 m–n.c.	427 (100%)	n.c.
El-Messidi et al., 2015 [16]	D	7,916,388	Invasive Hodgkin’s	638 (0.081)	9 m–n.c.	638 (100%)	n.c.
Ives et al., 2005 [26]	P	33,000 ^b^	Breast ^d^	148 (0.024 ^f^)	9 m–13 m	49 (33.1%)	99 (66.9%)
O’Meara et al., 2005 [8]	D	4,846,505	Invasive Melanoma	412 (0.085)	9 m–12 m	145 (35.2%)	263 (63.8%)
Rodriguez et al., 2008 [9]	D	4,846,505	Invasive Breast	797 (0.164)	9 m–12 m	179 (23.5%)	610 (76.5%)
Shechter Maor et al., 2018 [17]	D	11,846,300	Invasive Breast	772 (0.065)	9 m–n.c.	772 (100%)	n.c.
Yasmeen et al.,2005 [10]	D	4,846,505	Invasive Thyroid	595 (0.14)	9 m–12 m	129 (21.7%)	466 (78.3%)

D: deliveries; LB: live births; P: pregnancies; n.c. = not considered; n.r. = not reported; p. = pregnancy; a. = abortion. ^a^ Excluded nonmelanoma skin cancer. ^b^ Approximate. ^c^ At least 20-week gestation or 400 g birthweight. ^d^ Invasive and not invasive. ^e^ A further 146 cases were found but not included in the study. ^f^ Age standardized rate.

## Supplementary Materials

The following are available online at https://www.mdpi.com/2072-6694/12/6/1356/s1, Table S1: More frequent cancers identified. Studies on pregnancy-associated cancer (PAC) overall, Table S2: Evolution during years of the frequency of pregnancy-associated cancer (PAC). All studies that assessed trend over time.
