# Supplementary material for: Frequency of Pregnancy-Associated Cancer: A Systematic Review of Population-Based Studies
Source: Cancers (Basel). 2020 May 26;12(6):1356. doi: 10.3390/cancers12061356 (PMC7352408; doi:10.3390/cancers12061356)
Supplement: Supplementary file 1 [file cancers-12-01356-s001.pdf]

*Supplementary Materials*

# Frequency of Pregnancy Associated Cancer: A Systematic Review of Population-Based Studies

**Michela Dalmartello, Eva Negri, Carlo La Vecchia, Giovanna Scarfone, Barbara Buonomo, Fedro A. Peccatori and Fabio Parazzini**

**Table S1.** More frequent cancers identified. Studies on pregnancy associated cancer (PAC) overall.

| Author | More Frequent Types of Cancer (NR Cases) From Studies on PAC Overall | | |
|---|---|---|---|
| | **1st Cancer** | **2nd Cancer** | **3rd Cancer** |
| EU | | | |
| Andersson et al., 2015 [18] | Breast (1486) | Melanoma (1035) | Cervix (912) |
| Eibye et al., 2013 [20] | Melanoma (507) | Cervix (493) | Breast (489) |
| Lambe et al., 1995 [21] | Breast (248) | Cervix (278) | Melanoma (271) |
| Lu et al., 2017 [22] | Breast (707) | Melanoma (692) | Cervix (500) |
| Murgia et al., 2019 [23] | Breast (257) | Thyroid (152) | Skin ex melanoma (89) |
| Parazzini et al., 2017 [24] | Breast (479) | Thyroid (186) | Lymphoma (157) |
| USA | | | |
| Cottreau et al., 2019 [14] | Breast (208) | Thyroid (168) | Melanoma (93) |
| Smith et al., 2001 [4] | Breast (423) | Thyroid (389) | Cervix (266) |
| Smith et al., 2003 [5] | Breast (935) | Thyroid (699) | Cervix (580) |
| AU | | | |
| Lee et al., 2012 [2] | Melanoma (599) | Breast (377) | Thyroid & endocrine (228) |
| Lee et al., 2013 [3] | Melanoma (322) | Breast (218) | Thyroid & endocrine (133) |

n.r. = not reported.

**Table S2.** Evolution during years of the frequency of pregnancy associated cancer (PAC). All studies that assessed trend over time.

| Author | Length of Observation (Years) | Increase Over Time | Results |
|---|---|---|---|
| | | **EU** | |
| Eibye et al., 2013 [20] | 30 | YES | Annual percentage increase in incidence rate by 5 years, from 2.9% (95%CI: 2.4–3.3). After age adjustment: 1.6% (95% CI: 1.1–2.1) |
| Murgia et al., 2019 [23] | 13 | NO | |
| Parazzini et al., 2017 [24] | 12 | NO | |
| Andersson et al., 2009 (BREAST) [19] | 40 | YES | Rate increased from 0.16 to 0.374 from 1963 to 1974. After stratifying by age, there was an increase from 1963–1974 to 1975–1989 in all age groups. Incidence remained stable/decreased somewhat between the period 1975–1989 and 1990–2002. |
| | | **USA** | |
| Cottreau et al., 2019 [14] | 13 | YES | Increase between 2002–2012 from 0.75 (95% CI: 0.549–1.00) to 1.38 (95% CI: 1.09–1.733) |
| Smith et al., 2001 [4] | 6 | NO | |
| Abenhaim et al., 2012 (BREAST) [12] | 10 | NO | |
| Al-Halal et al., 2012 (CERVIX) [13] | 10 | NO | |
| Dalrymple et al., 2005 (CERVIX) [7] | 9 | NO | |
| El-Messidi et al., 2014 (NON-HODGKIN'S) [15] | 9 | YES | Represented in graphic ($p$-value = 0.001) |
| El-Messidi et al., 2015 (HODGKIN'S) [16] | 9 | NO | |
| O'Meara et al., 2005 (MELANOMA) [8] | 9 | NO | |
| Shechter Maor et al., 2018 (BREAST) [17] | 14 | YES | Increase between 1999 and 2012 ($p < 0.05$) with the incidence reaching 0.075 in 2012) |
| | | **AU** | |
| Lee et al., 2012 [2] | 15 | YES | Increase between 1994 and 2007 from 1.12 to 1.915 ($p$-vale < 0.001); only 14% of increase was explained increasing mother age |
| Bannister-Tyrrell et al., 2014 (MELANOMA) [25] | 15 | YES | Increase between 1994 and 2008 from 0.371 to 0.5184 |

CI = confidence interval.

© 2020 by the authors. Licensee MDPI, Basel, Switzerland. This article is an open access article distributed under the terms and conditions of the Creative Commons Attribution (CC BY) license (http://creativecommons.org/licenses/by/4.0/).
